# A strategy for mechanically integrating robust hydrogel-tissue hybrid to promote the anti-calcification and endothelialization of bioprosthetic heart valve

**DOI:** 10.1093/rb/rbae003

**Published:** 2024-01-30

**Authors:** Haoshuang Wu, Nuoya Chen, Tiantian Zheng, Li Li, Mengyue Hu, Yumei Qin, Gaoyang Guo, Li Yang, Yunbing Wang

**Affiliations:** National Engineering Research Center for Biomaterials, Sichuan University, Chengdu 610065, China; National Engineering Research Center for Biomaterials, Sichuan University, Chengdu 610065, China; National Engineering Research Center for Biomaterials, Sichuan University, Chengdu 610065, China; Institute of Clinical Pathology, West China Hospital of Sichuan University, Chengdu 610041, China; College of Polymer Science and Engineering, Sichuan University, Chengdu 610065, China; National Engineering Research Center for Biomaterials, Sichuan University, Chengdu 610065, China; National Engineering Research Center for Biomaterials, Sichuan University, Chengdu 610065, China; National Engineering Research Center for Biomaterials, Sichuan University, Chengdu 610065, China; National Engineering Research Center for Biomaterials, Sichuan University, Chengdu 610065, China

**Keywords:** bioprosthetic heart valves, recombinant humanized collagen type III, endothelialization, anti-calcification, anti-coagulation

## Abstract

Bioprosthetic heart valve (BHV) replacement has been the predominant treatment for severe heart valve diseases over decades. Most clinically available BHVs are crosslinked by glutaraldehyde (GLUT), while the high toxicity of residual GLUT could initiate calcification, severe thrombosis, and delayed endothelialization. Here, we construed a mechanically integrating robust hydrogel-tissue hybrid to improve the performance of BHVs. In particular, recombinant humanized collagen type III (rhCOLIII), which was precisely customized with anti-coagulant and pro-endothelialization bioactivity, was first incorporated into the polyvinyl alcohol (PVA)-based hydrogel via hydrogen bond interactions. Then, tannic acid was introduced to enhance the mechanical performance of PVA-based hydrogel and interfacial bonding between the hydrogel layer and bio-derived tissue due to the strong affinity for a wide range of substrates. *In vitro* and *in vivo* experimental results confirmed that the GLUT-crosslinked BHVs modified by the robust PVA-based hydrogel embedded rhCOLIII and TA possessed long-term anti-coagulant, accelerated endothelialization, mild inflammatory response and anti-calcification properties. Therefore, our mechanically integrating robust hydrogel-tissue hybrid strategy showed the potential to enhance the service function and prolong the service life of the BHVs after implantation.

## Graphical Abstract

**Figure:**
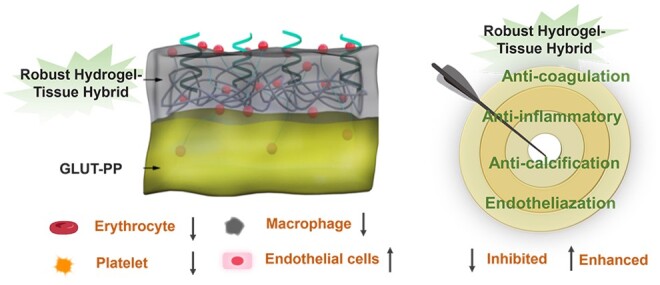

## Introduction

With the accelerated aging of the population, valvular heart diseases have become a severe threat to global human health, and it is expected to expand to 850 000 in 2050 [1]. Transcatheter aortic heart valve replacement, benefiting from not requiring thoracotomy, minimal risk and faster recovery, is considered the most effective and widely used treatment for heart valve diseases [2, 3]. At present, the clinical application of bioprosthetic heart valves (BHVs) has expanded rapidly, already surpassing mechanical heart valves [4, 5]. Most of the clinically available glutaraldehyde (GLUT) BHVs-crosslinked BHVs exhibit a limited lifespan after implantation, usually less than 15 years, and cannot meet the clinical demands ideally [1, 6–10]. The failure of GLUT-crosslinked BHV implantations mainly arises from thrombogenicity [11], cytotoxicity [12], severe immune responses and calcification [13]. The collagen exposed on the GLUT-crosslinked BHVs surface could stimulate the adhesion and activation of platelets, thereby causing acute thrombosis [14]. The high toxicity of residual GLUT can retard endothelialization, which is not favorable for improving the biocompatibility of blood-contacting devices after implantation [15]. Both calcification of BHVs, which is associated with residual free aldehyde groups [16], and severe immune responses incurred by poor biocompatibility could accelerate the GLUT-crosslinked BHVs failure. Therefore, a uniform and continuous covering surface with properties of excellent anti-coagulation and anti-calcification is an effective strategy for the functional modification of BHVs. It is widely accepted that reduced inflammatory responses and rapid re-endothelialization are prime modified directions for improving the performance of BHVs.

After BHVs implantation, a host reaction occurs, accompanied by the responses comprising fibrin adsorption, platelet adhesion and activation, followed by the initiation of the coagulation. The formation of subclinical leaflet thrombosis is potentially associated with late valve calcification, subsequently contributing to the service failure of leaflets. Various anticoagulant modification strategies have been applied to the blood-contacting surfaces, such as electrically neutral superhydrophilic coatings [17], zwitterionic polymers [18, 19], heparin [20] and NO-releasing/generating coatings [21]. However, these strategies are related to many potential side effects, such as instability (for heparin), toxicity (for NO) or preterm exhaustion [22]. In addition, the problem of partial and discontinuous surface modification could not be ignored, since the exposure of calcification sites on the GLUT-crosslinked BHVs could induce subsequent calcification behaviors. A persistent chronic inflammatory response might contribute to endothelialization disorder. Therefore, the modified BHVs were endowed with excellent anti-inflammatory and rapid endothelialization for prolonging the service life of BHVs.

Recently, soft hydrogel with tunable physicochemical properties, such as polycaprolactone, polyvinyl alcohol (PVA) and polyethylene glycol, have been extensively utilized in various fields [23, 24]. Among these hydrogels, PVA-based hydrogels due to their similar properties to natural tissues and organs with superior softness, high hydrophilicity, favorable chemical stability and excellent biocompatibility, have received considerable researchers’ attention [25, 26]. However, current applications of the single PVA hydrogel in biomedical fields are often limited by unfavorable mechanical performance and poor bioactivity [27, 28]. To improve the mechanical properties of PVA hydrogel and interfacial bonding between the hydrogel layer and bio-derived tissue, the introduction of binding agents is warranted. Due to the robust attachment capability to a wide range of substrates, marine mussels have been commonly utilized as a multifunctional platform, both as binding agents and a secondary platform for the introduction of additional functionalities [29]. Based on the above considerations, the tannic acid (TA) was incorporated and expected, on the one hand, to improve the mechanical properties of the PVA hydrogel itself as well as the interfacial bonding between the hydrogel layer and substrates. On the other hand, it offered the feasibility for follow-up loading of more bioactive molecules [17].

The recombinant humanized collagen type III (rhCOLIII) was thoughtfully customized via advanced genetic engineering and structural biology technologies based on our requirements. Different from the classical animal-derived collagens, the rhCOLIII possessed favorable water solubility, facilitated cell adhesion, anti-coagulant properties and reduced immunogenicity owing to the preservation of the high-adhesion segments of humanized collagen type III (Gly–Glu–Arg and Gly–Glu–Lys) as well as the circumvention of hydroxyproline (O) sequence that might initiate the adhesion and activation of platelets, and the removal of the immune-terminal peptides at the two ends of the collagen molecule—the N-terminus and the C-terminus [30, 31]. Furthermore, it also exhibited good water solubility. The above-mentioned characteristics of rhCOLIII open up avenues for its application in cardiovascular materials.

**Figure 1.**
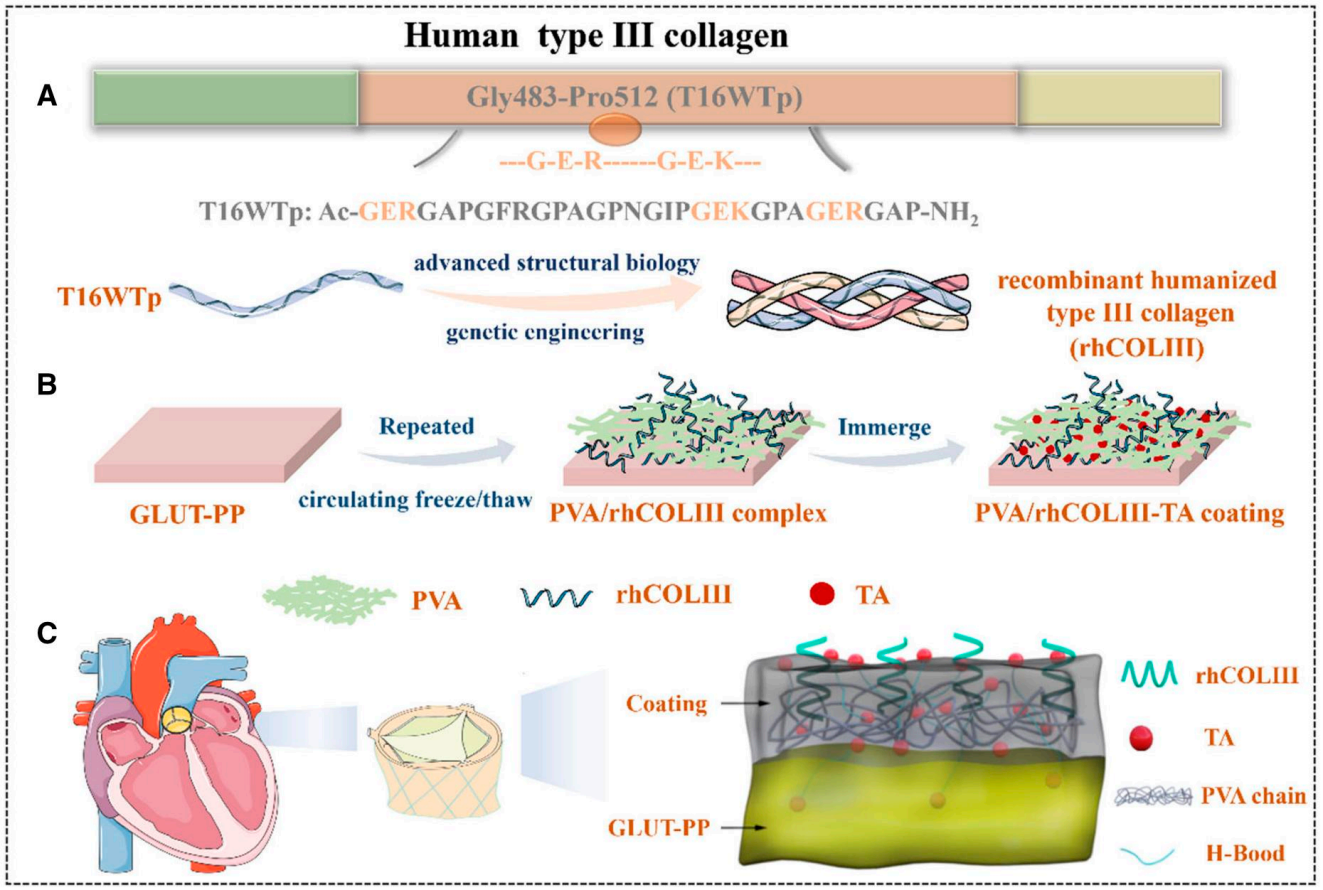
(**A**) Synthesis route of the rhCOLIII. (**B**) Preparation process of the mechanically robust integrated hydrogel-tissue embedded anticoagulant rhCOLIII and anti-inflammatory TA. (**C**) Structure of the BHVs modified with PVA/rhCOLIII-TA coating.

Herein, a mechanically robust integrated hydrogel-tissue embedded rhCOLIII and TA was proposed, which provided sustained anticoagulant, anti-inflammatory, rapid endothelialization and anti-calcification capacities. The rhCOLIII was meticulously tailored (Figure 1A), and the construction of the mechanically robust integrated PVA hydrogel-tissue consisting of two steps was shown in Figure 1B. The first step was to prepare the PVA/rhCOLIII complex network by circulating freeze/thaw method. The PVA/rhCOLIII hydrogel could form a uniform and continuous surface to cover calcification sites, endowing the BHVs with excellent anti-coagulant and anti-calcification capabilities. In the second step, the anti-inflammatory TA was introduced, which could significantly reinforce the integration between the hydrogel layer and BHVs leaflets, in addition to the formation of hydrogen bonds with rhCOLIII. With the synergistic contribution of PVA/rhCOLIII and TA, the PVA/rhCOLIII-based hydrogel coating with the incorporation of TA was expected to possess anti-coagulation, rapid endothelialization, anti-inflammatory and anti-calcification properties (Figure 1C). The chemical structure, mechanical properties, anti-coagulation properties, cytotoxicity, immune response and calcification were investigated *in vivo* or *in vitro*. This strategy provided a potential platform for improving the performance of cardiovascular implant/interventional materials not constrained to biological valves in terms of blood compatibility, re-endothelialization, anti-inflammation and anti-calcification.

## Materials and methods

### Materials

The porcine pericardia harvested from healthy experimental pigs were obtained from Venus MedTech Inc. (Hangzhou, China), and the porcine pericardium used in this study was from the same batch. rhCOLIII was kindly provided by Shanxi Jinbo Bio-pharmaceutical Co., Ltd. TA and PVA were purchased from Sigma-Aldrich Co. Ltd. The biological reagents used in this study would be referenced in the appropriate experimental section.

### Preparation of PVA/rhCOLIII-TA coating

Briefly, the porcine pericardia were immersed in GLUT solution at a concentration of 0.625% (v/v) with shaking for 7 days, and then GLUT-PP was obtained [32]. After that, GLUT-PP was rinsed with ultrapure water (DI) for further use.

The PVA/rhCOLIII-TA coating-modified GLUT-PP was fabricated by electrostatic interaction, hydrogen bonding and covalent bonding among the rhCOLIII, TA and PVA. Briefly, the GLUT-crosslinked heart valves were immersed in PVA solution (10% w/w in PBS) equal in volume to the rhCOLIII solution (2 mg/ml in PBS) for 1 h. Then, the modified heart leaflets were rinsed with DI water and freeze/thaw three cycles, and the product was referred to as PVA/rhCOLIII. The procedure of cycling/freezing was performed according to the mentioned in the previous literature [33]. The PVA/rhCOLIII-TA coating was obtained by immersing PVA/rhCOLIII modified heart valves in 1 mg/ml TA solution (pH = 7.4) for 24 h. During the preparation of the modified heart valves, the surface area of the valves to the volume ratio of the reaction liquid was 1:1. Notably, the GLUT-PP modified with PVA and TA, without rhCOLIII was named PVA-TA. GLUT-PP was used as the control.

### Mechanical testing

#### Uniaxial tensile test

The GLUT-PP, PVA-TA and PVA/hCOLIII-TA modified BHVs were cut into 3 mm × 15 mm rectangular strips, and then the specimens were mounted on the tensile tester (Bio-Tester 5000, Cellscale, California). Before testing, the specimens were stored in PBS solution and their average thickness was measured for stress calculation. The specimens were stretched at a constant velocity of 12.5 mm/min until they fractured. At the same time, the stress–strain curve of the samples was recorded for the calculation of ultimate tensile strength (UTS), tensile modulus and elongation.

#### Suture pull-out test

The GLUT-PP and modified BHVs were trimmed into rectangular shapes (15 mm*10 mm) and stored in PBS solution at room temperature for future use. The samples were secured on a clamp under the guidance of a nylon suture (3-0) by passing through the center of the samples. Subsequently, the samples were stretched until they were fractured, with the same procedure as in the uniaxial tensile experiments. The tear strength of the samples was calculated by the peak load (*F*), thickness (*T*), and width (*D*) [34].

#### In vitro simulation of stent expansion

The modified BHVs were stitched to the stents and then were dilated using a balloon in PBS solution at 37°C, simulating the process of angioplasty, and the binding of the coatings to the substrate was investigated by the morphology difference of the coating before and after stent expansion by SEM.

### Coating characterization

Before the test, the heart valves were sliced and then freeze-drying. Scanning electron microscopy (SEM, JEOL, JSM-7500F, Japan) was used to investigate the surface micromorphology of the samples. Atomic Force Microscope (AFM, Bruker) using tapping mode with a ScanAsyst-Air (Triangular) cantilever was utilized to evaluate the roughness of the heart leaflets. The surface chemical composites of all heart leaflets before and after modification were measured by ATR-FTIR (Spectrum One, Nicolet) and X-ray photoelectron spectroscopy (XPS, XSAM600, UK), respectively.

### Hemocompatibility

All animal experiment procedures in this study were approved by the Medical Ethics Committee of Sichuan University and were implemented strictly under the Guidelines for Care and Use of Laboratory Animals of Sichuan University (No. KS2020394). Rabbit whole blood was centrifuged at 5000 rpm for 15 min for red blood cells (RBCs), 1500 rpm for 15 min for platelet-rich plasma (PRP) and 3000 rpm for 15 min for platelet-poor plasma.

#### Determination of hemolysis rate

Before the test, RBCs suspension was prepared by mixing the RBCs with PBS at a ratio of 1:10. In addition, DI and PBS were used as positive and negative controls, respectively. The valve leaflets were incubated with RBCs suspension at 37°C for 2 h, and then the supernatant was collected and detected by a microplate reader.

$$\mathrm{Hemolysis}\;\mathrm{ratio}=\frac{{\mathrm{Absorbance}}_{\mathrm{Sample}}}{{\mathrm{Absorbance}}_{\mathrm{DW}}}\times 100\%$$

#### Protein adsorption

Before the test, GLUT-PP, PVA-TA and PVA/rhCOLIII-TA modified BHVs were immersed in PBS for 12 h for use. Different samples were then incubated with FITC-BSA solution (1 mg/ml) for 2 h at 37°C. After that, the samples were removed and gently rinsed three times with UP water. Protein adsorption on the sample surface was visualized by fluorescence image from a confocal laser scanning microscope (CLSM, Leica SP5, Germany).

#### Platelet adhesion test

The GLUT-PP and modified BHVs were incubated with PRP with a shaker at 37°C for 1 h. Afterward, the samples (*n* = 6) were rinsed three times with sterile PBS to remove non-adherent platelets. Quantification of adherent platelets was determined with the LDH kit (Shanghai BeoTeam Biotechnology Co., Ltd) following the supplier’s instructions. The morphology of platelet adhesion and activation on sample surfaces was qualitatively observed by SEM. The detailed information was shown in Supplementary Method S1.

#### Ex vivo antithrombogenicity assay

The animal experiments mentioned in this work were performed in a sterile environment. Before the test, GLUT-PP, PVA-TA and PVA/rhCOLIII-TA modified BHVs were carefully affixed to the internal lumen of the PVC tube. After 1 h of blood circulation, specimens were removed and fixed with paraformaldehyde overnight. The anticoagulant properties of the specimens were evaluated by observing the microscopic morphology of thrombi adhered to the surface of the GLUT-PP and modified BHVs using SEM [18]. The detailed information was shown in Supplementary Method S2.

### Cytotoxicity

The cytotoxicity assay was carried out strictly following ISO 10993-5:2009. Briefly, the samples (diameter = 8 mm) were immersed in Dulbecco’s modified eagle’s medium (DMEM) with 10% FBS and 1% penicillin/streptomycin for 3 days in an incubator with 5% CO_2_ at 37°C, and then the supernatant was extracted for the cytotoxicity test. Mouse fibroblasts (L929) suspension at the density of 2.0 × 10^4^ cells/ml was cultured with samples for 3 days. The old medium was replaced with 100 µl of extract each day during the incubation period. Cell activity was determined by a CCK-8 assay kit. Furthermore, live and dead cells after 3 days of incubation were colored with calcein and propidium iodide (PI) staining, respectively, and visualized with a fluorescence microscope (Nexcope NIB900, USA).

### Cell culture

The primary HUVECs and macrophages (RAW 264.7) were cultured with DMEM containing 10% fetal bovine serum and 1% penicillin/streptomycin in an incubator with 5% CO_2_ at 37°C.

#### Cell proliferation and morphology

The cell suspensions with a density of 2.0 × 10^4^ cells/ml (HUVECs or macrophages) were incubated with GLUT-PP, PVA-TA and PVA/rhCOLIII-TA samples in 48-well cell culture plates. After 3 and 7 days of incubation, the proliferation of cells on the surface of different samples was evaluated by a Cell Counting Kit-8 (CCK-8 Kit). The morphology of cells adherent to the samples was stained by fluorescein diacetate (FDA) and visualized by CLSM. In terms of HUVECs, a western blot (WB) assay was performed to characterize the growth state by analyzing the expression of HUVECs markers, including von Willebrand factor (vWF), interleukin-1β (IL-1β), endothelial nitric oxide synthase (eNOS) and platelet endothelial cell adhesion molecule-1 (CD31) [20, 35]. In addition, the polarization and the secretion of inflammatory factors of RAW 264.7 were quantified using flow cytometry (LSRFortessa SORP (BD)) and enzyme-linked immunosorbent assay (ELISA) kits, respectively (Supplementary Method S3) [36, 37].

### Stability tests

The stability of the heart valve specimens was evaluated using a peristaltic pump with a flowing system containing a PBS solution at 37°C. The device provided a stable flow rate of 20 ml/min and the PBS solution changed every day. Of note, the PVA/rhCOLIII-TA coating fabricated using FITC-labeled rhCOLIII was employed for the evaluation of structural stability. After being cycled for 3, 7, 15, and 30 days, respectively, the samples were removed and then the LDH assay and CCK8 test as mentioned above were performed to quantitatively evaluate the number of adhered platelets and the proliferation of HUVECs. Moreover, the *ex vivo* arteriovenous shunt assay and the fluorescent staining of HUVECs were conducted to investigate the long-term antithrombotic properties and the development of HUVECs of the modified valve leaflets 30 days after circulation.

### Subcutaneous implantation assay

Before this experiment, the GLUT-PP, PVA-TA and PVA/rhCOLIII-TA-modified heart valves were sterilized overnight. Firstly, male Sprague Dawley (SD) rats (*n* = 6, ∼50 g) were anesthetized with pentobarbital (25 mg/ml) by intraperitoneal injection. Then, the dorsal subcutaneous tissue of the rats was separated from the muscle using curved forceps, and specimens were carefully implanted in place of the subcutaneous tissue. After 15-day and 30-day implantation, the specimens with the surrounding capsule were removed from the rats and fixed in 10% paraformaldehyde to evaluate the degree of inflammation.

### Histological and immunohistological assay

H&E staining was applied to evaluate the tissue affinity of the specimens *in vivo*. The macrophage and T cells were marked with CD68 antibody (Anti-CD68 Rabbit, Bioss) and CD3 antibody (Anti-CD3 Rabbit, Bioss), respectively. After 90-day subcutaneous implantation, the samples were stained with Alizarin Red to evaluate calcification depositions. At least three random view fields were imaged for each sample, and the cell number was quantitively analyzed by Image J software.

### *In vitro* pulsatile flow testing and fatigue performance

First, BHVs wrapped with a stent were prepared by suturing the PVA/rhCOLIII-TA-coated BHVs to the stent. Then, the hydrodynamic properties of the PVA/rhCOLIII-TA-coated BHVs were assessed at different physiologically equivalent aortic pressures and blood flow conditions, with the effective orifice area (EOA) calculated using a pulsatile flow instrument. This experiment was performed following ISO 5840 guidelines, which were detailed in Supplementary Method S4.

PVA/rhCOLIII-TA-coated BHVs were submitted to accelerated fatigue testing following ISO 5840 guidelines using a fatigue instrument (AWT-1000, MITL, China). After 1 × 10^7^ cycles of the simulated clinical valve on/off cycling, we evaluated the fatigue performance of the modified BHV by evaluating its damage status.

### Statistical analysis

Statistical analyses for significant differences between different groups were performed by Student’s *t*-test and one-way analysis of variance. Analytical data are in the form of mean ± standard deviation. The star indicates a significant difference between GLUT-PP, PVA-TA, and PVA/rhCOLIII-TA (*P < 0.05, **P < 0.01, *** *P* < 0.001, and ****P <* *0.0001).

## Results and discussion

### Chemical structure characterizations

**Figure 2.**
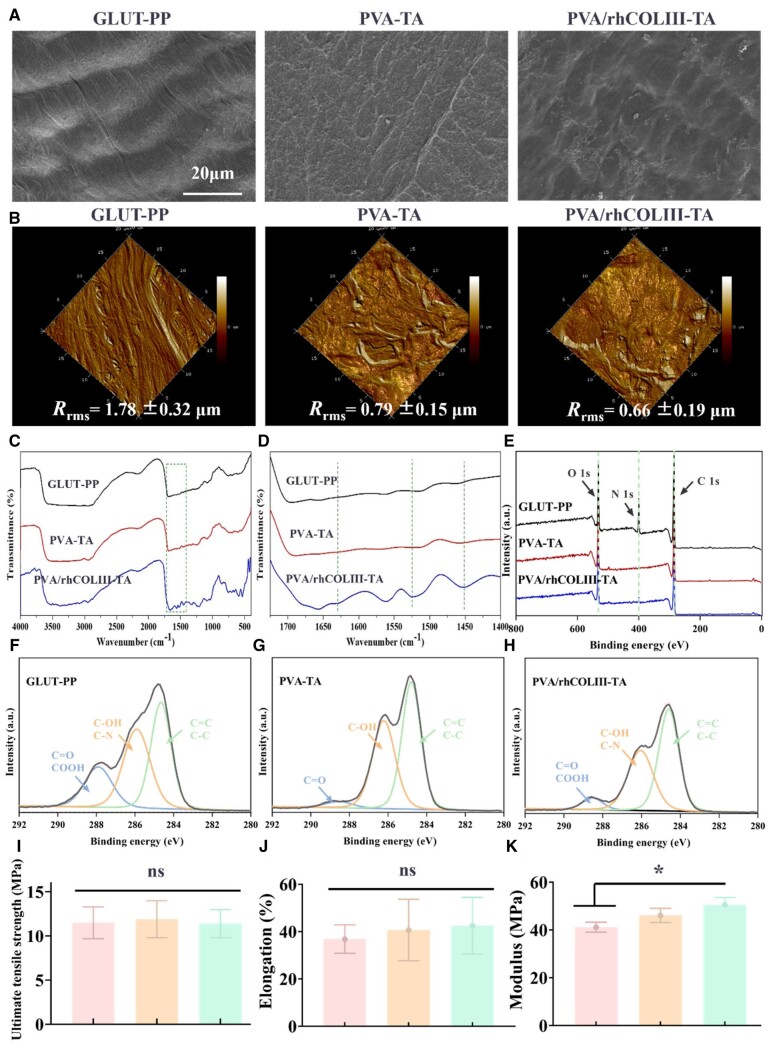
(**A**) SEM Images of GLUT-PP, PVA-TA and PVA/rhCOLIII-TA modified biological valve leaflets. Scale bars = 20 μm. (**B**) AFM images of GLUT-PP, PVA-TA and PVA/rhCOLIII-TA. (**C**) ATR-FTIR spectra of different samples. (**D**) Enlarged view of ATR-FTIR spectra. (**E**) Wide scan XPS spectra before and after modified BHVs. (**F–H**) Corresponding high resolution of the C1s spectrum of different samples. (**I**) UTS, (**J**) elongation and (**K**) tangent modulus of different samples. *n* = 6.

Variations of the surface micromorphology of the heart valve specimens before and after being modified were observed by SEM and AFM. As shown in Figure 2A, the fibrous structure is observed on the valve surfaces with large pores and uneven distribution. After modification with PVA-TA coating, the fibrous structure was invisible and a film-like substance appeared on the surface of biological valves. The PVA/rhCOLIII-TA coating presented a smoother texture compared with the GLUP-PP and PVA-TA groups, which was attributed to the formation of various forces between the hydroxyl groups of PVA and the carboxyl groups and hydroxyl groups of rhCOLIII, thereby enabling the network to be smoother and more stable. AFM data (Figure 2B) demonstrated that there was a gradual decrease in the surface roughness of PVA-TA (0.79 ± 0.15 μm) and PVA/rhCOLIII-TA (0.66 ± 0.19 μm) compared to the GLUP-PP (1.78 ± 0.32 μm) due to the shading of the fibrous structures, consistent with the results of SEM.

The surface molecular structure and chemical bonds of the modified valve were first verified by the ATR-FTIR spectrum analysis. As shown in Figure 2C and D, the peak near 1230 and 1380 cm^−1^ in the spectra of PVA/TA and PVA/rhCOLIII-TA groups were ascribed to the symmetric vibration of hydroxyl groups, whereas the new double peak near 2900 and 2850 cm^−1^ could be considered as the carboxylate stretching vibration of TA. The above results indicated the successful introduction of PVA and TA [38]. Furthermore, in comparison with PVA/TA, the peaks observed around 1525 and 1630 cm^−1^ in PVA/rhCOLIII-TA were attributed to the –COOH and –NH stretching vibration in rhCOLIII, respectively, implying the successful introduction of rhCOLIII [39]. The XPS full spectra and the high-resolution C1s spectra results also indicated the successful construction of the PVA/TA and PVA/rhCOLIII-TA coatings. In detail, the disappearance of the N1s peak and the increased ratio of O/C in the PVA-TA group (31.2%) compared to the GLUP-PP group (21.2%) provided strong evidence that PVA-TA coating was successfully attached to the surface of GLUP-PP (Figure 2E and Supplementary Figure S1). The successful incorporation of rhCOLIII was further evidenced by the re-appearance of the N1s peaks and the enhancement in the intensity of the COOH peak (288.3 eV) in the PVA/rhCOLIII-TA group in comparison to the PVA-TA (Figure 2F–H).

### Mechanical property

The BHVs in service are required to possess good mechanical properties. The maintenance of good mechanical properties is beneficial to the physiological function of the valve after implantation. UTS was performed to indicate the maximum load of samples. The PVA-TA and PVA/rhCOLIII-TA-modified biological valve leaflets showed no significant difference in UTS and elongation, compared with the GLUT-PP (Figure 2I and J). The tangent modulus (50.6 ± 3.0 MPa) and tearing strength (4.38 ± 1.04 MPa) of the PVA/rhCOLIII-TA group were improved in comparison with those of the GLUT-PP group (41.2 ± 2.1 and 2.68 ± 0.3 MPa, respectively), but there was no significant difference (Figure 2K and Supplementary Figure S2). To furthermore realistically examine the mechanical stability of the PVA-TA and PVA/rhCOLIII-TA modified BHVs, we observed the surface morphology of different samples before and after stent dilation by SEM. As shown in Supplementary Figure S3A and B, no obvious separation and tears were observed in PVA-TA and PVA/rhCOLIII-TA after stent extension, indicating that the coatings possessed favorable bonding with the native BHVs and were able to fulfill the requirements of the interventional delivery mode. These results indicated that the modification of PVA-TA and PVA/hCOLIII-TA had not dramatically affected the mechanical properties of GLUT-crosslinked BHVs.

### Hemocompatibility

**Figure 3.**
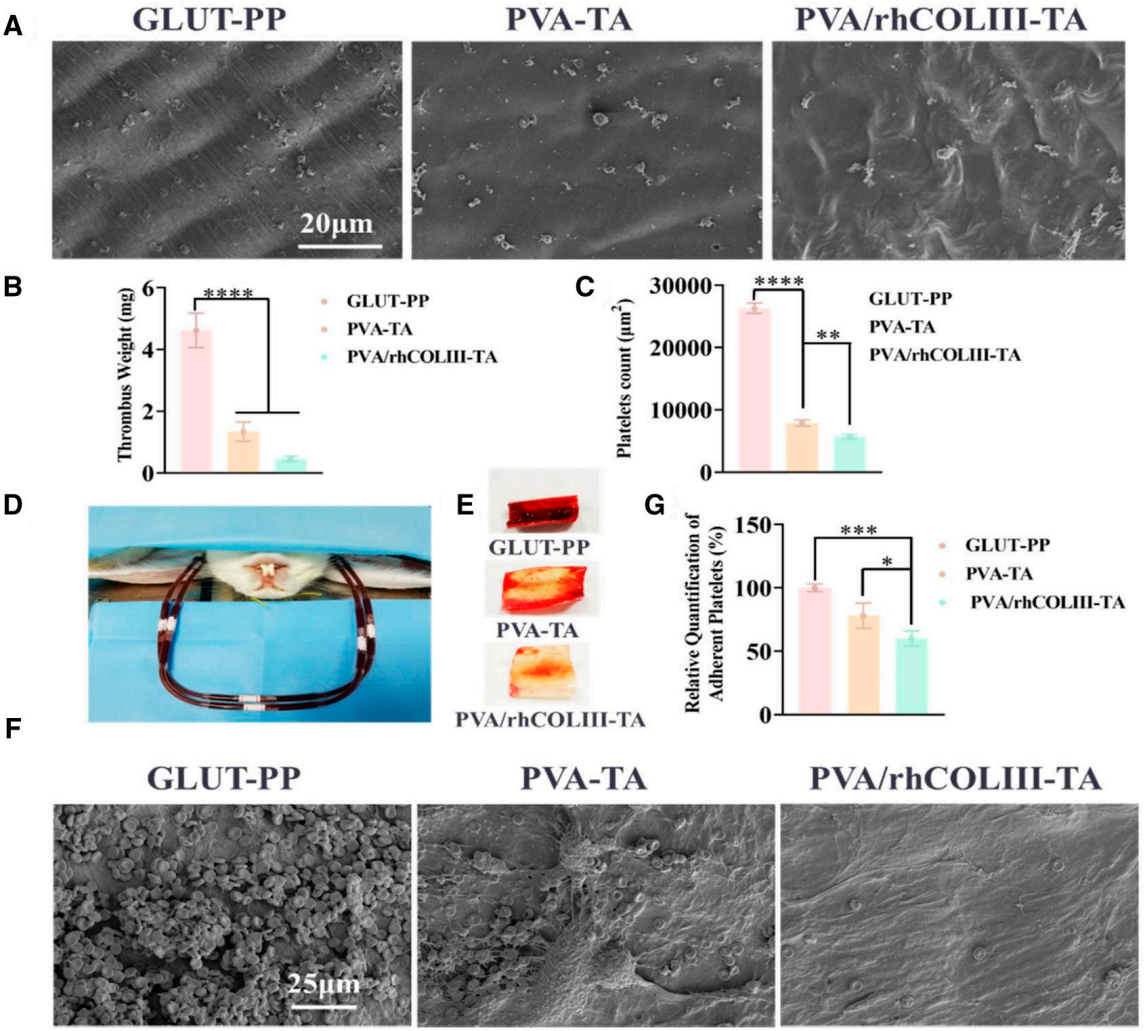
(**A**) Platelet adhesion and activation on GLUT-PP, PVA-TA and PVA/rhCOLIII-TA-modified heart valve specimens, observed by SEM. (**B**) The relative adhesion of platelets was measured by LDH kit on the different sample surfaces. (**C**) Corresponding quantitative analysis of platelet counts on different samples. (**D**) Illustration of the arteriovenous shunt rabbit model *ex vivo*. (**E**) Photographs of thrombus on valve leaflets surfaces. (**F**) SEM images and (**G**) weights of the surface thrombus before and after BHVs modification after 1 h of cycling.

The antithrombotic properties of the modified BHVs were assessed by *in vitro* protein adsorption and platelet adhesion assays, and *ex vivo* arteriovenous shunt assays [40, 41]. As shown in Supplementary Figure S4, the PVA/hCOLIII-TA-modified BHVs significantly inhibited protein adsorption compared with other groups. Consistent with this, abundantly activated platelets with pseudopods were observed on the surface of the GLUT-PP group (Figure 3A). In contrast, only a small number of inactivated round platelets were discovered on the PVA/rhCOLIII-TA surface, probably associated with the excellent anticoagulant behavior of rhCOLIII. In addition, the quantitative LDH and platelet adhesion number results (Figure 3B and C) also confirmed that there were much fewer platelets adhered in the PVA-TA and the PVA/rhCOLIII-TA groups than in the control group. The anti-platelet activation function of the PVA-TA coating was further improved compared to the control, which might contribute to the covering of platelet binding sites of heart valve leaflets. Supplementary Figure S5 showed that the hemolysis rates of GLUT-PP, PVA-TA and PVA/rhCOLIII-TA were 1.93 ± 0.15%, 1.23 ± 0.21% and 0.92 ± 0.08%, respectively, which demonstrated that the hemolysis rate of the BHVs modified with PVA/rhCOLIII-TA was lower than that of GLUT-crosslinked BHVs samples. The rate of hemolysis under 5% is considered reliable and safe according to medical standards [42].

A rabbit arteriovenous shunt test *ex vivo* was carried out to assess the antithrombotic properties of the modified BHVs more realistically after implantation (Figure 3D). After 1 h of blood circulation, the GLUT-PP surface was covered by a severe thrombosis with activated platelets, fibrin and erythrocytes aggregation (Figure 3E and F). The PVA-TA-modified BHVs displayed a small amount of platelet adhesion and lower activation levels. No thrombus or platelets appeared on the PVA/rhCOLIII-TA modified heart leaflets. Besides, the thrombus weight in PVA/rhCOLIII-TA was clearly lower than the other two groups (GLUT-PP and PVA-TA) (Figure 3G). To sum up, PVA/rhCOLIII-TA performed the most excellent anticoagulant properties and demonstrated potential in improving the blood compatibility of cardiovascular implant/interventional materials.

### Cytocompatibility

**Figure 4.**
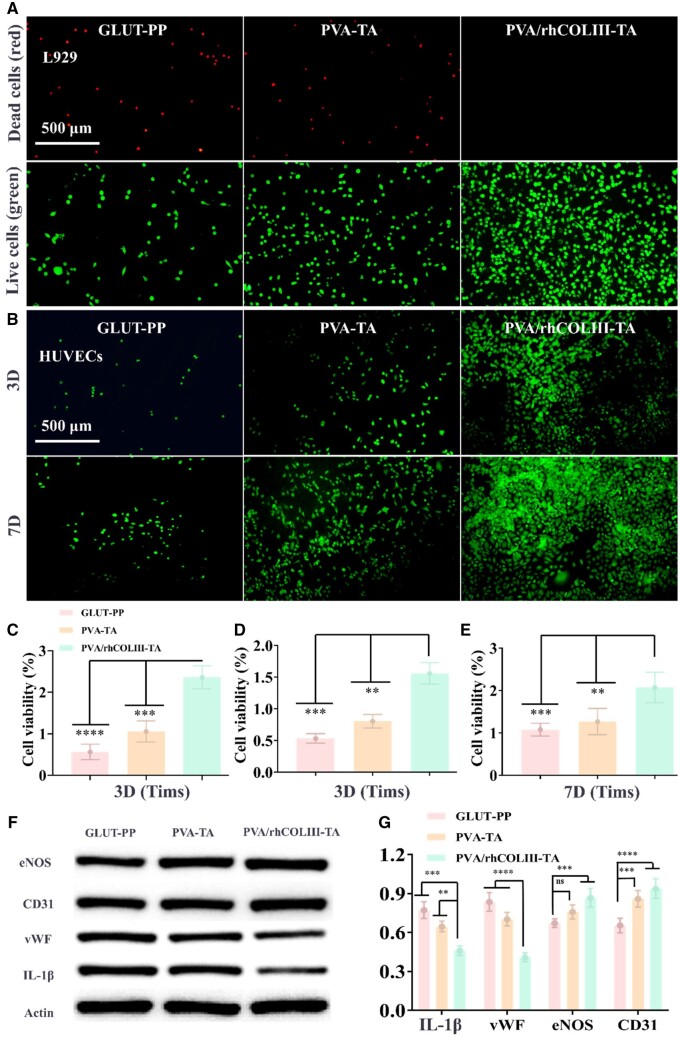
(**A**) The live/dead cells were labeled with calcein and PI after incubation with GLUT-PP, PVA-TA and PVA/rhCOLIII-TA extracts for 3 days. (**B**) Fluorescent staining of the HUVECs on GLUT-PP, PVA-TA and PVA/rhCOLIII-TA-modified heart valve leaflets after incubation for 3 and 7 days at 37°C, respectively. (**C**) Cell viability of L929 after 3 days of culturing. Cell viability of HUVECs on the different valve leaflets for (**D**) 3 and (**E**) 7 days. (**F**) Characteristic WB bands of HUVECs-related proteins and (**G**) quantitative analysis of 1L-1β, vWF, eNOS and CD31.

The cytotoxicity of modified BHVs was evaluated by incubating L929 cells with extracts from different samples for 3 days [43, 44]. As shown in Figure 4A, more live cells and barely any dead cells were observed in the PVA/rhCOLIII-TA group compared to the other two groups (GLUT-PP and PVA-TA). Moreover, the cell viability of PVA/rhCOLIII-TA-modified BHVs was significantly higher than the other two groups (Figure 4C), which corroborated with the live/dead staining results. All these results suggested that the extracts of PVA/rhCOLIII-TA were virtually free of cytotoxicity to L929 cells.

Rapid re-endothelialization promotes the integration between material and host tissue and effectively avoids material failure triggered by late thrombosis and late restenosis of vascular implants, which is a common requirement of most cardiovascular implants [45, 46]. First, the proliferation of HUVECs was analyzed by qualitative FDA staining and quantitative metabolic CCK-8 assay, respectively. After 3 days of culturing, poor HUVECs adhesion and proliferation were detected in the GLUT-PP group owing to the GLUT cytotoxicity (Figure 4B). Notably, the cell number and coverage of the PVA/rhCOLIII-TA group were obviously higher than the other two groups, suggesting a healthy and actively developing state of HUVECs [47]. As the incubation time was prolonged to 7 days, the capacity of PVA/rhCOLIII-TA in facilitating cell adhesion and proliferation was more prominent compared to the GLUT-PP and PVA-TA groups, as evidenced by the significant increase in the cell number and the healthy spindle endothelial-like morphology. Moreover, the results of CCK-8 assays (Figure 4D and E) also confirmed the phenomenon above, especially the cell viability of PVA/rhCOLIII-TA was approximately 2-fold higher than that of PVA-TA after 7 days of culturing. Encouraged by the above-mentioned positive results, a WB assay was performed to further investigate the mechanisms of PVA/rhCOLIII-TA promoting HUVECs growth. As shown in Figure 4F and G, the expression of eNOS and CD31 were clearly upregulated by PVA/rhCOLIII-TA in comparison to other groups, while 1L-1β and vWF were downregulated. These results demonstrated that the PVA/rhCOLIII-TA-modified BHVs facilitated HUVECs’ growth while maintaining high endothelial function, which provided the basis for rapid endothelialization. Overall, the PVA/rhCOLIII-TA-modified BHVs exhibited better cytocompatibility than GLUT-PP and PVA-TA probably attributed to the fact that, in addition to the function of TA in promoting cell adhesion and proliferation, rhCOLIII could also enhance the adhesion and proliferation of cells via numerous cell binding sites.

### *In vitro* anti-inflammation evaluation

**Figure 5.**
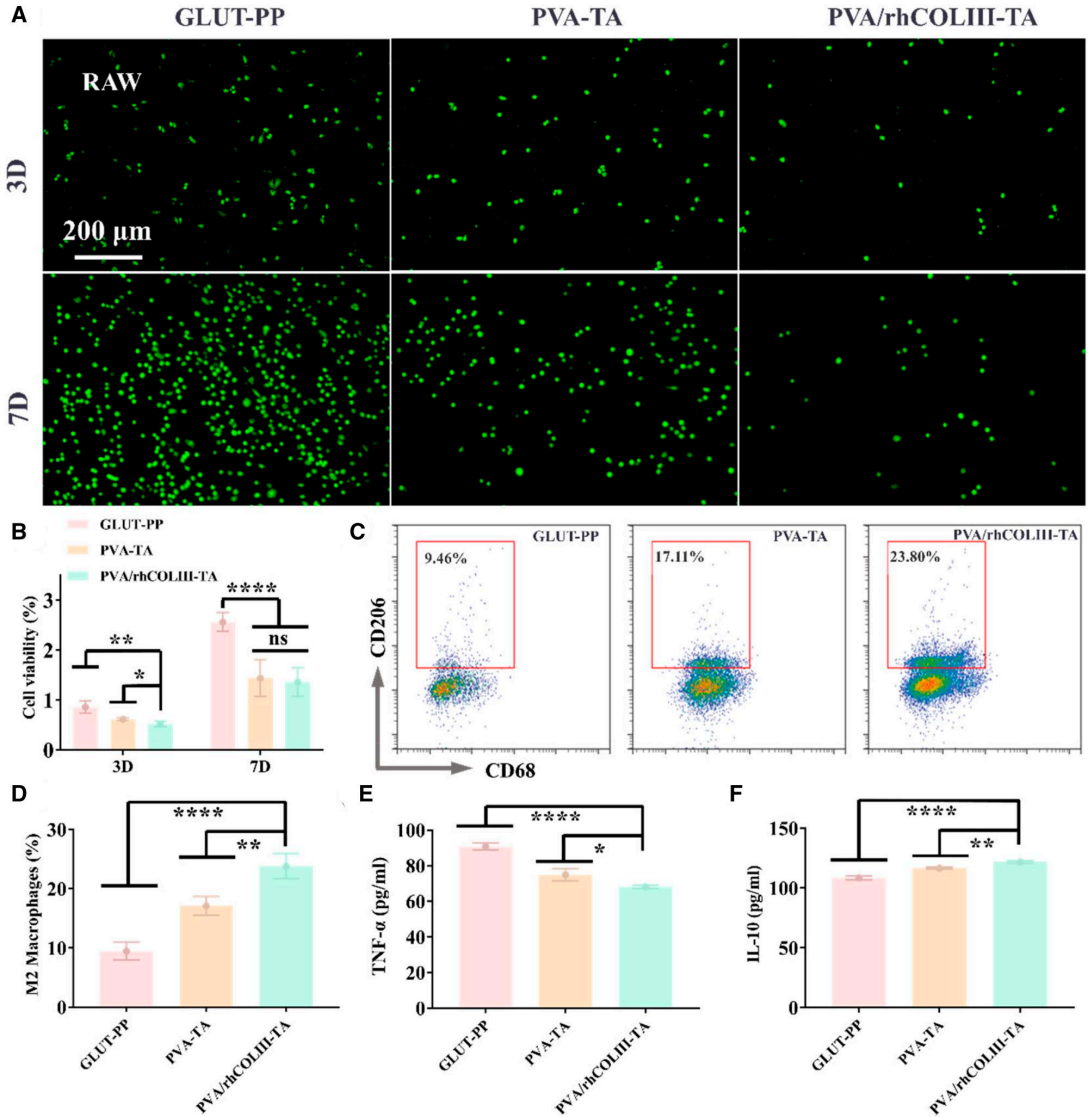
(**A**) Fluorescent staining of the RAW 264.7 on GLUT-PP, PVA-TA and PVA/rhCOLIII-TA-modified heart valve leaflets for 3 and 7 days at 37°C, respectively. (**B**) Cell viability of different samples. (**C**) Representative flow cytometry plots of M2 phenotypic RAW 264.7 after different sample treatments. (**D**) Quantification of M2 phenotypic RAW 264.7. (**E**) TNF-α and (**F**) IL-10 levels expressed by RAW 264.7 detected by ELISA.

The mild cellular inflammatory tissue responses after sample implantation favor a prolonged service of biological valve leaflets, while the severe inflammatory response could induce subsequent valve calcification [48]. After co-incubation with the samples for 3 days, the morphology of RAW 264.7 on the surface of the samples was visualized by fluorescence staining. A large number of RAW 264.7 with irregular shapes were observed in the control group (Figure 5A). In contrast, there was only a small quantity of macrophages adhered in the PVA-TA and PVA/rhCOLIII-TA, and most of them were round in a dormant state. With the extension of the incubation time, the count and activation level of RAW 264.7 in the GLUT-PP group were significantly aggravated, whereas the macrophages in the PVA/rhCOLIII-TA group remained dormant and were the lowest in number. Meanwhile, the cell activity results (Figure 5B) were consistent with those of fluorescence staining results. Next, M2 phenotypic macrophages characterized by the CD206 marker were quantified by flow cytometry. As shown in Figure 5C and D, only 9.46% of M2-polarization of macrophage cells was observed in GLUT-PP groups, whereas it gradually increased to 17.11% and 23.80% in the PVA-TA and PVA/rhCOLIII-TA groups, respectively. At the same time, the anti-inflammatory cytokine IL-10 and pro-inflammatory cytokines TNF-α were also detected to assess the anti-inflammatory behavior of different groups. Specifically, lower expression of TNF-α and higher expression of IL-10 were observed in the PVA/rhCOLIII-TA group compared to the GLUT-PP control group (Figure 5E and F). These results suggested that the PVA/rhCOLIII-TA coating surface could effectively inhibit the adhesion and proliferation of macrophages and had a potential anti-inflammatory effect.

### Stability test

**Figure 6.**
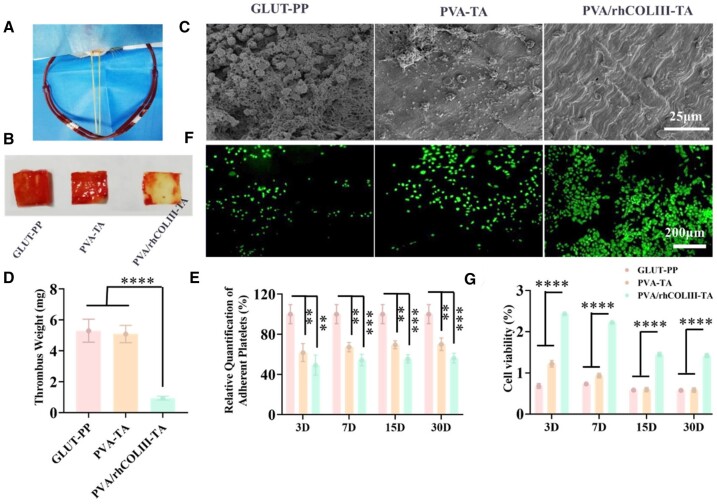
Stability of coatings modified biological valve leaflets after immersion in PBS at designed times. (**A**) Illustration of the arteriovenous shunt rabbit model *ex vivo*. (**B**) Photographs of thrombus on valve leaflets surfaces. (**C**) SEM images and (**D**) weights of thrombus formed on GLUT, PVA-TA and PVA/rhCOLIII-TA-modified biological valve leaflets after immersion for 30 days in PBS at 37°C after 1 h of cycling. (**E**) Relative quantification adhesion of platelets after 3D, 7D, 15D and 30 D of immersion in PBS solution at 37°C, determined by the LDH kit. (**F**) Fluorescent staining of different samples cultured with HUVEC for 3 days at 37°C after 30 days of immersion in PBS solution at 37°C. (**G**) Cell viability of different valve leaflets cultured with HUVEC for 3 days after 3D, 7D, 15D, and 30D of immersion in PBS solution at 37°C.

The functional stability of implanted heart valve specimens treated with PVA-TA and PVA/rhCOLIII-TA coatings was related to the service time. As shown in Supplementary Figure S6A and B, the fluorescence intensity of PVA/rhCOLIII-TA-modified BHVs was 93.7%, 87.7%, 78.5, %and 66.7% of that of fresh coating (fluorescence intensity was set to 100%) after circulation for 3D, 7D, 15D and 30D, respectively. These results confirmed the adequate stability of the PVA/rhCOLIII-TA, providing the basis for reliable biological functions for a longer time. Afterward, we proceeded with an *ex vivo* rabbit intravenous shunt assay to detect the long-term antithrombotic capability of the samples after 30 days of immersion in PBS (Figure 6A). Compared with the control GLUT-PP group, the PVA/rhCOLIII-TA group could effectively suppress the formation of thrombus. There were a large number of thrombi composed of fibronectin, erythrocytes, and activated platelets were discovered in the GLUT-PP and PVA-TA groups Impressively, almost no visible thrombus was observed in the PVA/hCOLIII-TA group (Figure 6B and C). The weight thrombosis of GLUT-PP and PVA-TA were approximately 5.3 ± 0.74 and 5.09 ± 0.56 mg, respectively. However, only a small amount of thrombus (0.94 ± 0.12 mg blood clot) occurred on the PVA/rhCOLIII-TA-modified biological valve leaflets (Figure 6D), and this corroborated the SEM results. Besides, the hemocompatibility stability of samples under physiological conditions was examined by the LDH test. As shown in Figure 6E, almost no significant difference in the anti-platelet adhesion properties of the PVA/rhCOLIII-TA coating was observed after incubating in PBS solution for 3D, 7D, 15D, and 30D, respectively. Particularly, the PVA/rhCOLIII-TA coating displayed the best anticoagulant effect among all groups. The above results confirmed the long-acting anticoagulant efficacy of the PVA/rhCOLIII-TA coating.

The modified BHVs exert anticoagulant and rapid endothelialization properties stably at the initial period of implantation, which is of great significance for implantation/interventional devices [49]. After incubating with PBS, the adhesion and proliferation observed on the surface of samples were analyzed by FDA staining and metabolic CCK-8 assay. Notably, cell adhesion and proliferation were significantly promoted in the PVA/rhCOLIII-TA group in comparison with the GLUT-PP and PVA-TA groups (Figure 6F). Moreover, the cell viability of both PVA-TA and PVA/rhCOLIII-TA decreased slightly with the prolongation of incubating time compared to the fresh coatings (Figure 6G). These results indicated that the PVA/rhCOLIII-TA coating exhibited long-term anticoagulant as well as rapid endothelialization capabilities, which were essential at the initial period of BHVs implantation.

### Hydrodynamic and fatigue performance

To further investigate the potential in practical applications of PVA/rhCOLIII-TA-modified BHVs, we executed pulsatile flow tests under conditions simulating normal cardiac physiology [50–52]. The EOA of the BHVs modified with PVA/rhCOLIII-TA coating was between 1.889 and 2.197 cm^2^ at different mean aortic pressures, exceeding the minimal EOA criterion (1.7 cm^2^) (Supplementary Figure S7A). In particular, the EOA of the PVA/rhCOLIII-TA ranged from 2.016 to 2.150 cm^2^ under the standard test conditions (beat rates of 70 beats/min, cardiac output of 5 l/min and aortic pressure of 100 mmHg), was higher than the maximal reference of ISO5840-3 (1.95 cm^2^). Meanwhile, the durability of the BHVs modified with PVA/rhCOLIII-TA coating was initially evaluated under the guidance of the ISO5840-3 standard. The valve leaflets were validated to open and close normally without any structural damage after 1 × 10^7^ effective cardiac cycles (Supplementary Figure S7B). Of note, no phenomena involving incomplete opening and closing, fracture detachment, tearing or perforation were observed in PVA/rhCOLIII-TA-modified BHVs during the testing process. This preliminary promising result encourages us to accomplish subsequent, more thorough investigations.

### *In vivo* inflammatory response

**Figure 7.**
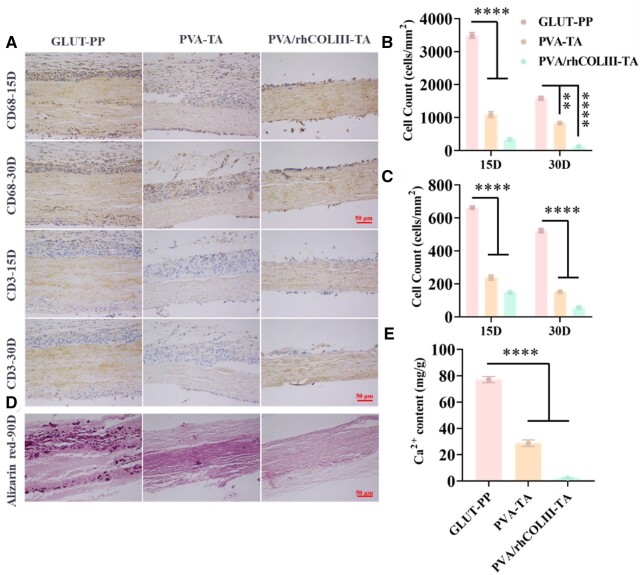
(**A**) Immunohistochemistry CD68 and CD3 staining of biological valve leaflets before and after modifications after implantation at 15 and 30 days. Quantitative analysis of the counts of (**B**) CD68-positive and (**C**) CD3-positive cells in different samples. (**D**) Alizarin red staining of GLUT-PP, PVA-TA and PVA/rhCOLIII-TA group slices. (**E**) Calcium contents of different samples after implantation for 90 days.

Host response of biological valve leaves *in vivo* could be evaluated by subcutaneous implantation assay in male SD rats [53, 54]. The degree of inflammatory response is positively correlated with the thickness of the surrounding fibrous capsule and the degree of infiltration of inflammatory cells [55]. The recruited macrophages and T lymphocytes around GLUT-PP, PVA-TA and PVA/rhCOLIII-TA-modified biological valve leaflets were observed after 15-day and 30-day implantation. As shown in Figure 7A and Supplementary Figure S8, the acute immune response in the GLUT-PP group was more severe compared to the PVA-TA and PVA/rhCOLIII-TA groups after 15-day implantation, as evidenced by thicker fibrous capsules and larger inflammatory cell infiltration. After 30 days of implantation, the most severe inflammatory response remained in the GLUT-PP group, whereas those in the PVA-TA and PVA/rhCOLIII-TA groups were further attenuated. The number of CD3-positive cells around all samples was much lower than that of CD68-positive cells all the time (Figure 7B and C), indicating the predominance of the non-specific immune response after implantation. The results indicated that PVA/rhCOLIII-TA could improve the host response of GLUT-crosslinked BHVs considerably both *in vitro* and *in vivo*.

### Calcification

Calcification is a predominant cause for the dysfunction of GLUT cross-linked BHVs, primarily caused by the binding of exposed aldehyde and carboxyl groups to calcium ions [56–58]. Furthermore, coagulation and inflammation could further increase the risk of calcification [59, 60]. As shown in Figure 7D, several red calcified sites were discovered in the GLUT-PP group, whereas no obvious calcified spots were detected in the PVA/rhCOLIII-TA group. Quantification of calcium amounts in different samples via inductively coupled plasma optical emission spectroscopy further confirmed that the PVA/rhCOLIII-TA group was effective in reducing calcification (Figure 7E). The severe calcification in the control group might be attributed to the residual cytotoxic aldehyde groups on the GLUT cross-linked BHVs and chronic inflammation, while the PVA-TA coating uniformly shielded the exposed toxic aldehyde groups, thereby reducing the degree of calcification. Interestingly, the PVA/rhCOLIII-TA coating presented more prominent anti-calcification properties compared to the PVA-TA coating, which was due to the synergistic contribution of the favorable anticoagulant, anti-inflammatory and pro-endothelial growth properties of rhCOLIII. In summary, PVA/rhCOLIII-TA exhibited long-term antithrombotic and anti-inflammatory capabilities and is a prospective candidate for optimizing the properties of GLUT cross-linked BHVs.

## Conclusion

In summary, a mechanically robust integrated hydrogel-tissue embedded rhCOLIII and TA strategy for promoting the anti-calcification and endothelialization of BHV was designed, which was composed of two modules. The primary module was a PVA/rhCOLIII hydrogel coating, which not only imparted superior softness but also provided a biocompatible interface to resist the formation of acute thrombosis and calcification. The second module was the TA, which not only reinforced the mechanical properties of the PVA-based hydrogel itself as well as the interfacial bonding between the hydrogel layer and substrate but also collaborated with rhCOLIII in improving the anti-inflammatory and rapid endothelialization of the GLUT-crosslinked BHVs. The effectiveness of PVA/rhCOLIII-TA in improving anticoagulation, suppressing inflammatory response and calcification and accomplishing accelerated re-reendothelialization was confirmed by *in vitro* and *in vivo* studies. All in all, this mechanically integrating robust hydrogel-tissue hybrid strategy may provide a promising direction in enhancing the service function and prolonging the service life of the BHVs after implantation.

## Supplementary Material

rbae003_Supplementary_Data
