# Supplementary material for: A strategy for mechanically integrating robust hydrogel-tissue hybrid to promote the anti-calcification and endothelialization of bioprosthetic heart valve
Source: Regen Biomater. 2024 Jan 30;11:rbae003. doi: 10.1093/rb/rbae003 (PMC10898858; doi:10.1093/rb/rbae003)
Supplement: rbae003_Supplementary_Data [file rbae003_supplementary_data.docx]

**A Strategy for Mechanically Integrating Robust Hydrogel-Tissue Hybrid to Promote the Anti-calcification and Endothelialization of Bioprosthetic Heart Valve**

Haoshuang Wu ^a^, Nuoya Chen ^a^, Tiantian Zheng ^a^, Li Li ^b^, Mengyue Hu ^c^, Yumei Qin ^a^, Gaoyang Guo ^a^, Li Yang ^a^, Yunbing Wang *^, a^

^a^ National Engineering Research Center for Biomaterials, Sichuan University, Chengdu 610065, China

^b^ Institute of Clinical Pathology, West China Hospital of Sichuan University, Chengdu, 610041, China.

^c^ College of Polymer Science and Engineering, Sichuan University, Chengdu 610065, China

*: Corresponding author

E-mail address: [yunbing.wang@scu.edu.cn](mailto:yunbing.wang@scu.edu.cn) (Y. Wang).

**Contents:**

**S1. Supporting Methods**

Method S1. Platelet adhesion test.

Method S2. *Ex vivo* antithrombogenicity assay.

Method S3. Flow cytometry and ELISA assays.

Method S4. *In Vitro* Pulsatile Flow Testing.

**S2. Supporting Figures**

Figure S1. Atomic percentages of elements at GLUT-PP, PVA-TA, and PVA/rhCOLIII-TA modified BHVs surface determined by XPS.

Figure S2. Tearing strength of GLUT-PP, PVA-TA, and PVA/rhCOLIII-TA modified BHVs. n=6.

Figure S3. (a) *In vitro* simulation of suture, compression and expansion of covered stent. (b) SEM images of PVA-TA and PVA/rhCOLIII-TA modified BHVs after stent dilation.

Figure S4. Fluorescence microscope images of adsorbed BSA-FITC and FBG-FITC on GLUT-PP, PVA-TA, and PVA/hCOLIII-TA.

Figure S5. Hemolyticability of GLUT-PP, PVA-TA, and PVA/rhCOLIII-TA groups.

Figure S6. (a) Surface fluorescence image of FITC-labeled PVA/rhCOLIII-TA in the flowing system with PBS solution for 3 D, 7 D, 15 D, and 30 D, respectively. (b) Quantification of fluorescence intensity.

Figure S7. Hydrodynamic and fatigue performance of the biological valve leaflets. (a)

EOA of the PVA/rhCOLIII-TA under different mean aortic pressures. (b) The photo

of the closed and open status of TA/Fe-rhCOLIII-modified BHVs.

Figure S8. Photographs of histomorphological (H&E) evaluation of GLUT-PP, PVA-TA, and PVA/rhCOLIII-TA subcutaneously implanted for 15 and 30 days.

**Method S1. Platelet adhesion test.**

The specimens of heart leaflets modified with GLUT-PP, PVA-TA, and PVA/rhCOLIII-TA (ϕ = 8 mm, n = 6) were placed in 48-well plates and incubated with PBS for 1 h. After that, the specimens were incubated in 200 μl PRP with a shaker at 37 ◦ C for 1 h. The PRP was discarded and the specimens were rinsed with PBS three times. For the SEM characterization, the specimens were fixed with 4% paraformaldehyde overnight and rinsed with PBS three times. The specimens were washed with a series of gradient alcohol solutions (60%, 70%, 80%, 90%, and 100%) for dehydration and were dried at room temperature. Qualitative and quantitative analyses of platelet adhesion and activation on sample surfaces were observed by SEM and LDH kits (Beyotime, Shanghai, China), respectively.

**Method S2. *Ex vivo* antithrombogenicity assay.**

The animal experiments mentioned in this work were performed in a sterile environment. Before the test, GLUT-PP, PVA-TA, and PVA/rhCOLIII-TA modified BHVs were carefully affixed to the internal lumen of PVC tube. Following that, the right carotid artery and left jugular vein of New Zealand white adult rabbits (male, ~2.5 kg) were isolated and joined to assembled tubes to develop an arteriovenous extracorporeal circulation circuit. After 1 h of blood circulation, specimens were removed and fixed with paraformaldehyde overnight. The anticoagulant properties of the specimens were evaluated by observing the microscopic morphology of thrombi adhered to the surface of the GLUT-PP and modified BHVs using SEM [1]. The detailed information was shown in Supporting Information (Method S2).

**Method S3. Flow cytometry and ELISA assays.**

Macrophages and their supernatant were collected 3 days after seeding onto six-well plates, respectively. In detail, M2-polarization and total of macrophages were labeled with PE-CD206 and FITC-CD68, respectively, with this procedure performed according to the supplier's guidelines, and then the polarization and the secretion of inflammatory factors of RAW 264.7 were quantified using flow cytometry LSRFortessa SORP (BD). Enzyme-linked immunosorbent assay (ELISA) (Jingmei Biotechnology) was used to detect the TNF-α and IL-10 levels of the collected supernatants.

**Method S4. In Vitro Pulsatile Flow Testing.**

First, the BHVs wrapped with a stent were prepared by suturing the PVA/rhCOLIII-TA-coated BHVs to the stent with a diameter of 29 mm. Then, the hydrodynamic properties of the PVA/rhCOLIII-TA-modified BHVs were assessed at different physiologically equivalent aortic pressures and blood flow conditions, with the effective orifice area calculated using an *in vitro* pulsatile flow instrument (Shanghai Heartpartner Testing Equipment Co., Ltd., China). This experiment was performed following ISO 5840 guidelines. Pulsating flow test parameters: heart rate 45– 120 /min, cardiac output 2–7 L/min, average pressure 80–160 mmHg, pressure difference 40 mmHg [2].


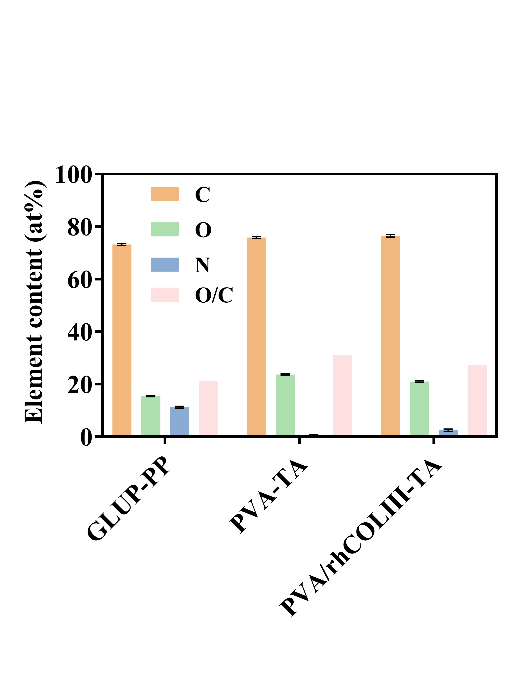


Figure S1. Atomic percentages of elements at GLUT-PP, PVA-TA, and PVA/rhCOLIII-TA modified BHVs surface determined by XPS.


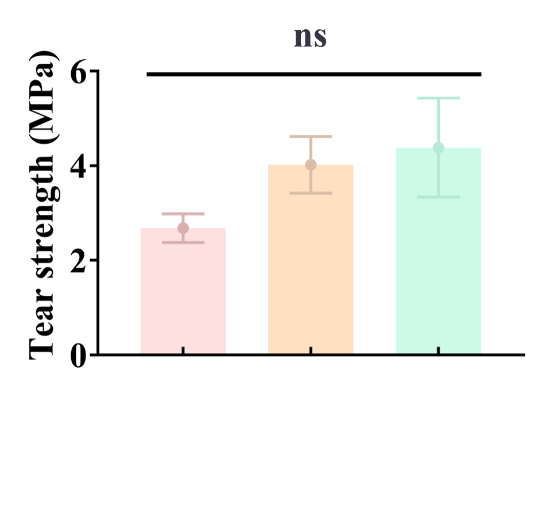


Figure S2. Tearing strength of GLUT-PP, PVA-TA, and PVA/rhCOLIII-TA modified BHVs. n=6.


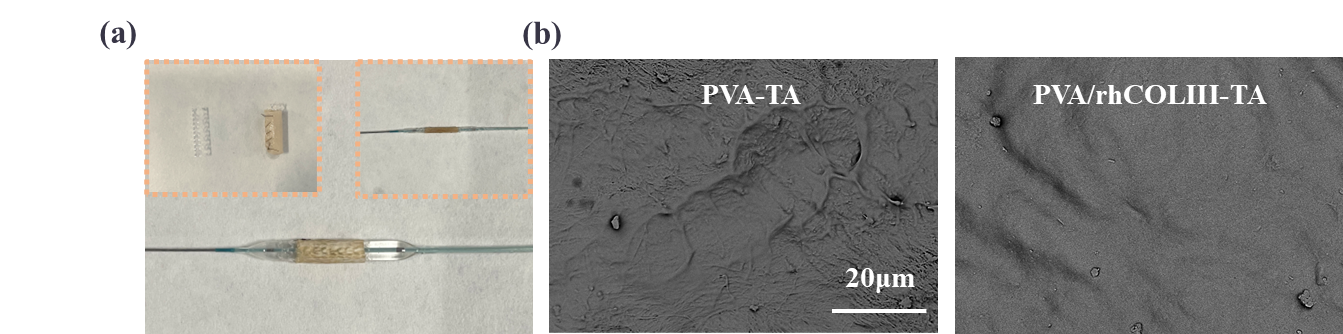


Figure S3. (a) *In vitro* simulation of suture, compression and expansion of covered stent. (b) SEM images of PVA-TA and PVA/rhCOLIII-TA modified BHVs after stent dilation.


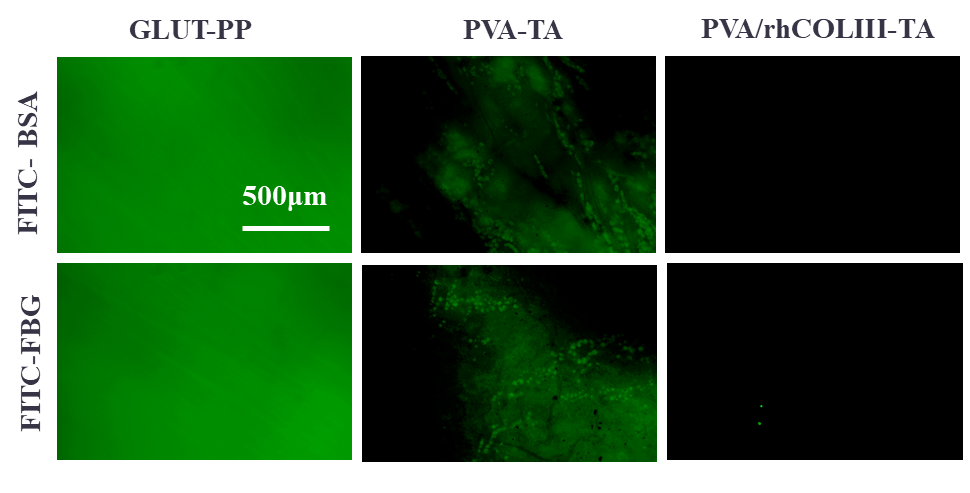


Figure S4. Fluorescence microscope images of adsorbed BSA-FITC and FBG-FITC on GLUT-PP, PVA-TA, and PVA/hCOLIII-TA.


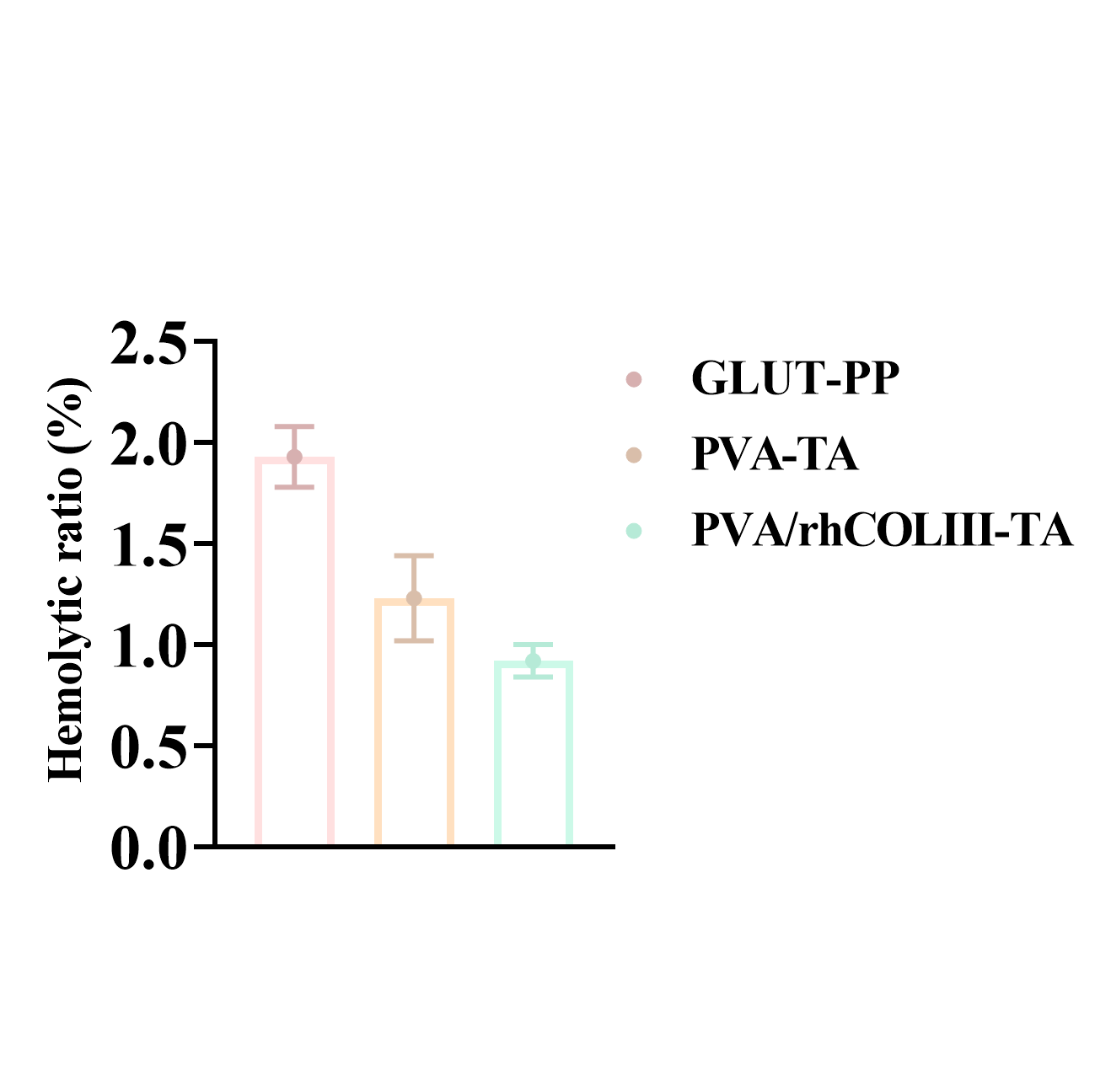


Figure S5. Hemolyticability of GLUT-PP, PVA-TA, and PVA/rhCOLIII-TA groups.


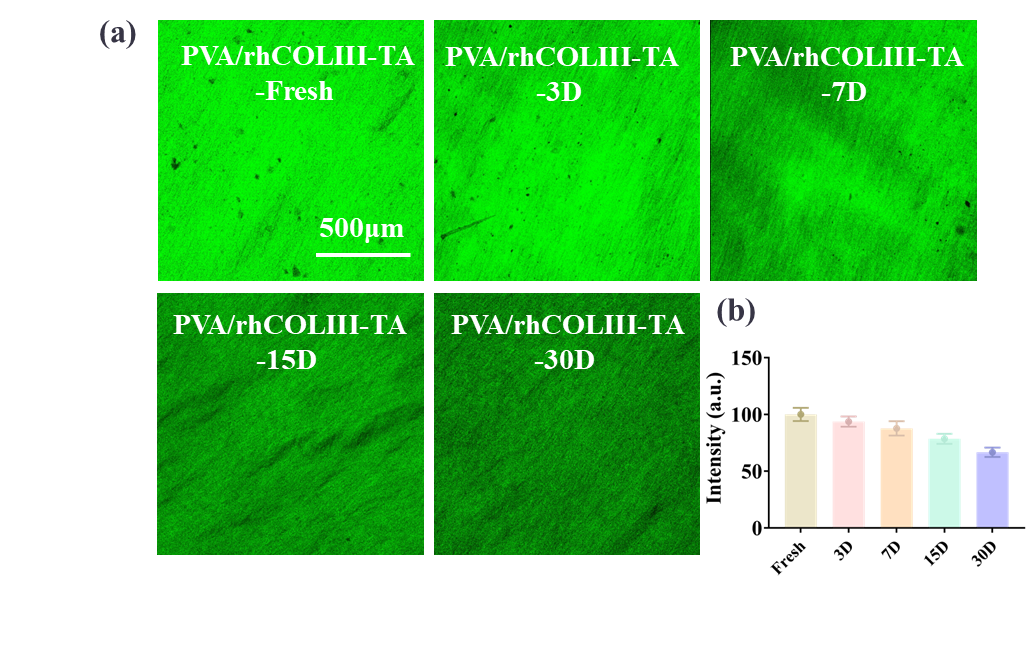


Figure S6. (a) Surface fluorescence image of FITC-labeled PVA/rhCOLIII-TA in the flowing system with PBS solution for 3 D, 7 D, 15 D, and 30 D, respectively. (b) Quantification of fluorescence intensity.


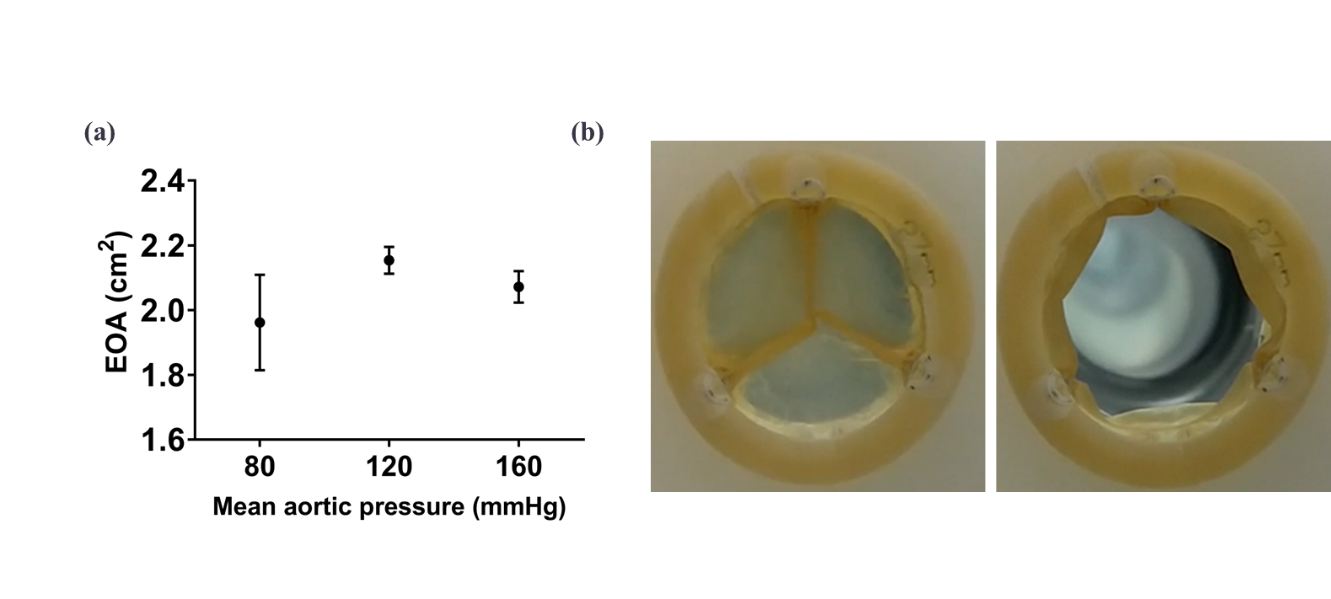


Figure S7. Hydrodynamic and fatigue performance of the biological valve leaflets. (a)

EOA of the PVA/rhCOLIII-TA under different mean aortic pressures. (b) The photo

of the closed and open status of TA/Fe-rhCOLIII-modified BHVs.


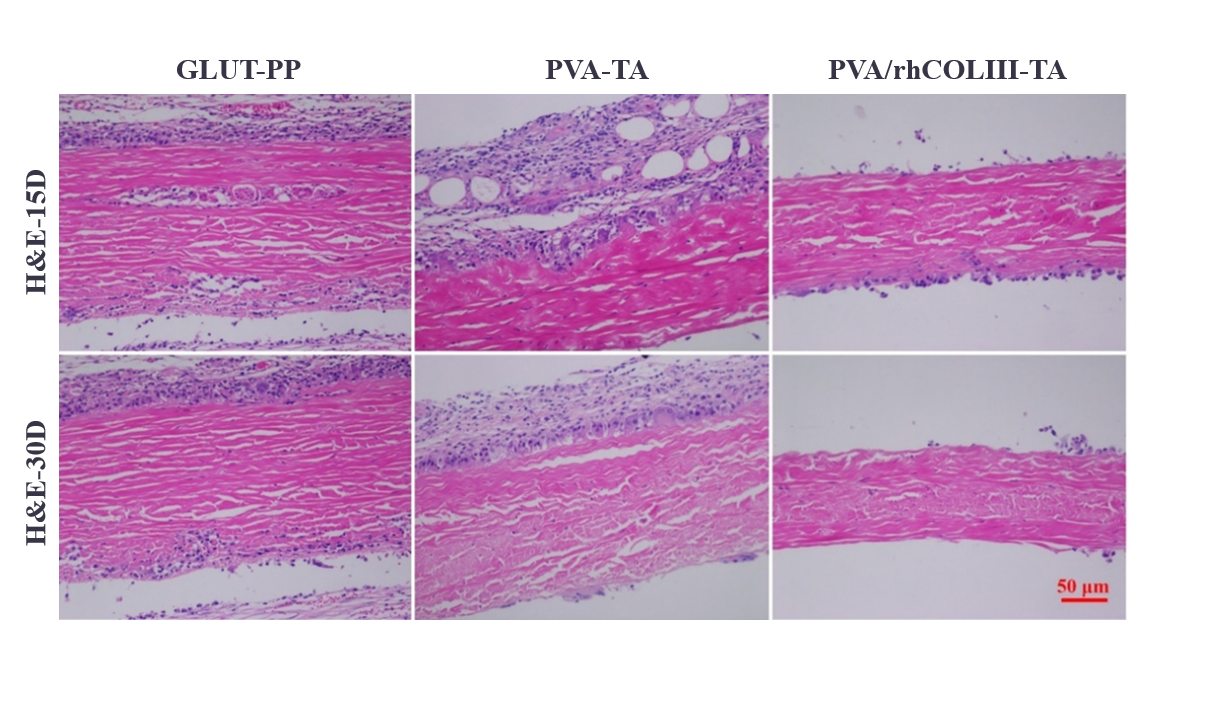


Figure S8. Photographs of histomorphological (H&E) evaluation of GLUT-PP, PVA-TA, and PVA/rhCOLIII-TA subcutaneously implanted for 15 and 30 days.

**Reference**

1. Zhang J, He L, Wei G, Jiang X, Fu L, Zhao Y, Zhang L, Yang L, Li Y, Wang Y, Mo H, Shen J. Zwitterionic Polymer-Grafted Polylactic Acid Vascular Patches Based on a Decellularized Scaffold for Tissue Engineering. *Acs Biomaterials Science & Engineering* 2019;5:4366-4375.

2. Yu T, Chen X, Zhuang W, Tian Y, Liang Z, Kong Q, Hu C, Li G, Wang Y, Nonglutaraldehyde treated porcine pericardium with good biocompatibility, reduced Page 43 of 44 Journal of Materials Chemistry B calcification and improved Anti-coagulation for bioprosthetic heart valve applications. *Chemical Engineering Journal* 2021;414:128900.
